# Supplementary material for: A fault-intrusion-tolerant system and deadline-aware algorithm for scheduling scientific workflow in the cloud
Source: PeerJ Comput Sci. 2021 Nov 2;7:e747. doi: 10.7717/peerj-cs.747 (PMC8576554; doi:10.7717/peerj-cs.747)
Supplement: Supplemental Information 6 [file peerj-cs-07-747-s006.zip › Myproject1.1/docs/org/cloudbus/cloudsim/network/datacenter/package-frame.html]

org.cloudbus.cloudsim.network.datacenter


# org.cloudbus.cloudsim.network.datacenter

## Classes

- AggregateSwitch
- AppCloudlet
- EdgeSwitch
- HostPacket
- NetDatacenterBroker
- NetworkCloudlet
- NetworkCloudletSpaceSharedScheduler
- NetworkConstants
- NetworkDatacenter
- NetworkHost
- NetworkPacket
- NetworkVm
- NetworkVmAllocationPolicy
- RootSwitch
- Switch
- TaskStage
- WorkflowApp
